# Functional Characterization of the Photosynthetic Machinery in *Smicronix* Galls on the Parasitic Plant *Cuscuta campestris* by JIP-Test

**DOI:** 10.3390/cells10061399

**Published:** 2021-06-05

**Authors:** Lyuben Zagorchev, Alexandra Atanasova, Ivanela Albanova, Anelia Traianova, Petko Mladenov, Margarita Kouzmanova, Vasilij Goltsev, Hazem M. Kalaji, Denitsa Teofanova

**Affiliations:** 1 Faculty of Biology, Sofia University “St. Kliment Ohridski”, 8 Dragan Tsankov Blvd., 1164 Sofia, Bulgaria; atanassova.y@gmail.com (A.A.); albanova.ivanela@gmail.com (I.A.); a.traianova11@gmail.com (A.T.); mkouzmanova@gmail.com (M.K.); goltsev@gmail.com (V.G.); teofanova@biofac.uni-sofia.bg (D.T.); 2 AgroBioInstitute, Agricultural Academy, 8 Dragan Tsankov Blvd., 1164 Sofia, Bulgaria; rubisko@abv.bg; 3 Institute of Biology, Warsaw University of Life Sciences–SGGW, 02–787 Warsaw, Poland; hazem@kalaji.pl; 4 Institute of Technology and Life Sciences-National Research Institute, Falenty, Al. Hrabska 3, 05-090 Raszyn, Poland

**Keywords:** chlorophyll fluorescence, insect galls, JIP-test, parasitic plants

## Abstract

Members of the genus *Cuscuta* are generally considered to be non-photosynthetic, stem-holoparasitic flowering plants. Under certain circumstances, at least some members of the genus are capable of limited photosynthesis. The galls of the *Smicronyx* weevils formed on *Cuscuta campestris* are particularly rich in chlorophylls compared to the stem of the parasitic plant. In the present study, we aimed to characterize the photosynthetic activity in the inner and outer gall cortices in comparison to the non-photosynthetic stems and a reference plant (*Arabidopsis thaliana*). The recorded prompt chlorophyll fluorescence transients were analyzed using JIP test. Detailed analysis of the chlorophyll fluorescence confirmed the presence of actively functioning photosynthetic machinery, especially in the inner cortex of the galls. This photosynthesis, induced by the insect larvae, did not reach the levels of the photosynthetic activity in *Arabidopsis thaliana* plants. Thylakoid protein complexes were identified by separation with two-dimensional Blue Native/SDS PAGE. It appeared that some of the complexes presented in *A. thaliana* are missing in *C. campestris.* We hypothesize that the insect-triggered transition from non-photosynthetic to photosynthetic tissue in the gall is driven by the increased requirements for nutrients related to the larval nutrition.

## 1. Introduction

*Cuscuta campestris* Yunck. (Convolvulaceae) is a stem-holoparasitic flowering plant with a broad host range. Originally from North America, it is currently distributed worldwide and considered a noxious weed [1]. A distinct characteristic of all members of the genus is their ability to acquire water, minerals, and organic nutrients through haustorial connection with the host’s vascular system [2]. Because of that, members of the genus do not have functional leaves—leaves are completely missing or reduced to scales, and the plants are considered non-photosynthetic. Various sources reported a partial loss of photosynthesis-related genes, both in the nuclear genome and in the plastome [3,4,5].

Unlike other members of the genus, including the subgenus *Grammica*, *C. campestris* retained substantial part of the plastome genes [6]. In fact, no gene losses were detected in the sets for photosystems I and II, cytochrome b_6_/f complex, ATP synthase, or RuBisCO large subunit, but most of the NADH dehydrogenase genes were lost in this particular species. Furthermore, the nuclear genome of *C. campestris* retained all genes, needed for chlorophylls and carotenoids biosynthesis and most of the genes, needed for the linear and cyclic electron transport in the light-dependent photosynthetic reactions [7]. Therefore, *C. campestris* seems to contain chloroplasts and chlorophyll (Chl) concentrations, sufficient for at least limited photosynthesis as compared to fully non-photosynthetic *C. odorata* and *C. grandiflora*, but slightly weaker than *C. reflexa* [8]. However, this plant does not actually photosynthesize or at least does not do it at rates sufficient to sustain growth. It is logical that under optimal conditions, e.g., suitable host, when nutrients are readily available, it is unnecessary for the parasite to spend resources for photosynthetic activity. The photosynthetic ability, however, seems to be fully retained and may be triggered by various factors such as adaptable features to meet suboptimal conditions.

One such trigger was recently established—epiparasites of the genus *Smicronyx* (Coleoptera: Curculionidae), gall-forming insects, which prefer *Cuscuta* spp. as hosts [9]. Unlike other galls on photosynthetic plants where photosynthesis seems to decrease in the gall tissues [10], *Smicronyx* galls on *C. campestris* revealed much higher photosynthetic activity than non-infected *Cuscuta* stems [11]. It was also found that the inner cortex around the larval chamber is much more abundant in chlorophylls than the outer cortex or the stem [12]. *Smicronix* epiparasites are considered as a potential means for biological control of their agriculturally harmful hosts [13]. Although several reports on *Smicronix* gall formation in *Cuscuta* spp. exist, the metabolic and functional changes, occurring in the galls, remained largely unknown.

In the present study, we aimed to do a more detailed analysis of the light-dependent photosynthetic reactions in *C. campestris* stem in both the inner and outer cortex as compared to a fully photosynthesizing reference plant *Arabidopsis thaliana*. *A. thaliana* PSA has been studied in detail, its structure is well known, and it is the same as in the other green plants. Further, the thylakoid protein complexes in and outside the galls were investigated. Thus, both the elusive *Smicronyx*–*Cuscuta* interactions and the photosynthetic potential of *C. campestris* were considered.

## 2. Materials and Methods

### 2.1. Plant Material

**Figure 1:**
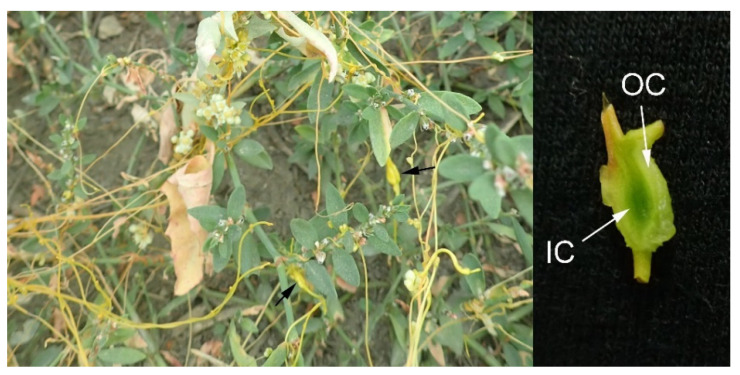
Yellowish stems of *Cuscuta campestris* on host *Polygonum aviculare* (**left panel**). Arrows indicate *Smicronyx* galls. Cross-section of a gall (**right panel**). The yellowish outer cortex (OC) and greenish inner cortex (IC) are indicated with arrows.

*Cuscuta campestris* plants with *Smicronyx* sp. galls were collected in late June from a wild population in the village of Telish, Cherven Briag municipality, Pleven province, the Danubian plain, Bulgaria (GPS 43°19′27.3″ N 24°15′15.8″ E). Plants were transferred with soil and with host plants together (mostly with *Polygonum aviculare* L. and *Convolvulus arvensis* L.) (Figure 1) into a greenhouse, adjacent to reference *Arabidopsis thaliana* plants. They were adapted for two days before further experiments. A voucher herbarium was deposited in the Herbarium SO (Sofia University “St. Kliment Ohridski”, Sofia, Bulgaria) under herbarium number SO 107784.

The reference *Arabidopsis thaliana* L. plants, ecotype Columbia (Col-0), were grown from seeds, obtained from the Nottingham Arabidopsis Stock Centre, in greenhouse conditions, under illumination with Kingbo KB-GLX45 Full Spectrum LED light. Approximately three-week-old plants were used in further experiments.

### 2.2. Chlorophyll a Fluorescence Measurement

The prompt Chl *a* fluorescence was measured using MPEA fluorometer (Multifunctional Plant Efficiency Analyzer, Hansatech Instruments Ltd., King’s Lynn, UK) after a dark-adaptation for 60 min. The PF measuring protocol included a 1 s pulse with high light intensity of 4000 µmol photons m^−2^ s^−1^. *Arabidopsis* leaves were measured in vivo. *Smicronyx* sp. galls were cut into 2 mm thick slices, and the corresponding inner and outer cortices were measured separately. *Cuscuta campestris* stems were also measured, but the intensity of the fluorescence signal was comparable to the background noise of the electronic device, so the results are not shown.

### 2.3. JIP-Test Parameters

JIP-test parameters were calculated from the obtained data for Chl *a* fluorescence based on the equations given by Strasser et al. [14] and Stirbet and Govindjee [15] and are presented in Table A1 in Appendix A.

### 2.4. Difference Curves Calculations

Every point on the double normalized curves of variable fluorescence was calculated by the equation (see Table A1):

V_t_ = (F_t_ − F_O_)/(F_M_ − F_O_) (1)

where V_t_ is a value of the relative variable fluorescence at moment t; F_t_—fluorescence level at that moment; F_O_—minimal fluorescence level; F_M_—maximal fluorescence level.

Each value of difference curves (DC) was calculated as a difference between the values of the relative variable fluorescence [V_t_ = (F_t_ − F_O_)/(F_M_ − F_O_)] calculated for the investigated gall cortex minus the respective values for the reference plant [ΔV_t_ = V_t_(gall) − V_t_(*A. thaliana*)].

DC for the characteristic phases of the induction curve (ΔW), calculated by subtracting the values of the IC for the control plants from the IC values of the stressed plants, are known as bands [14,15,16]. In this experiment, we calculated the differences in relative variable fluorescence values between gall cortices (inner or outer) and reference plant *A. thaliana*. There are four bands: L band (between O and 300 μs, ΔW_OK_), K band (between O and J, ΔW_OJ_), H band (between J and I, ΔW_JI_), and G band (between I and P, ΔW_IP_), and their positive or negative values determine the efficiency and rate of different reactions at every stage of the process. ΔW was calculated for each band by double normalization of the raw PF signal at specific steps as follows:

For O−K phase, L band:

W_t(OK)_ = (F_t_ − F_O_)/(F_K_ − F_O_); ΔW_OK_ = W_t(OK)(inner or outer cortex)_ − W_t(OK)(*A. thaliana*)_; (2)

For O−J phase, K band:

W_t(OJ)_ = (F_t_ − F_O_)/(F_J_ − F_O_); ΔW_OJ_ = W_t(OJ)(inner or outer cortex)_ − W_t(OJ)(*A. thaliana*)_; (3)

For J−I phase, H band:

W_t(JI)_ = (F_t_ − F_J_)/(F_I_ − F_J_); ΔW_JI_ = W_t(JI)(inner or outer cortex)_ − W_t(JI) (*A. thaliana*)_; (4)

For I−P phase, G band:

W_t(IP)_ = (F_t_ − F_I_)/(F_P_ − F_I_); ΔW_IP_ = W_t(IP)(inner or outer cortex)_ − W_t(IP) (*A. thaliana*)_. (5)

### 2.5. Statistical Analysis

A total of seven galls (outer and inner cortex), and leaves of five individual *A. thaliana* plants were used for chlorophyll fluorescence transients recording. Average values of the induction curves and standard errors were calculated from seven repetitions for all the investigated objects.

### 2.6. Thylakoid Protein Complexes Isolation and Separation

Thylakoid protein complexes were isolated and solubilized essentially by the method of Järvi [17]. Due to a plant material availability limitation, however, several steps were omitted or modified in order to prevent losses. The inner and outer cortices of individual galls were sliced, weighted (10 mg each), and grounded in liquid nitrogen. Equal weights of *Arabidopsis* leaves and *C. campestris* stems were processed in parallel, all under dim green light. The resulting powder was resuspended successively in ice-cold grinding, shock, and storage buffers with 10 mM NaF [17] and centrifuged at 5000× *g* at 4 °C after each buffer. Filtration through Miracloth was omitted. Finally, the pellets were solubilized in 20 µL 25 mM BisTris/HCl (pH 7.0) and 20% (*v/v*) glycerol; supplemented with 1% (*w/v*) digitonin; shaken at room temperature for 10 min; supplemented with 2 µL 100 mM BisTris/HCl (pH 7.0), 30% (*w/v*) sucrose, and 50 mg mL^−1^ Coomassie Blue G buffer; and centrifuged for 10 min at 16,000× *g*, and the whole amount was loaded onto Blue Native Polyacrylamide Gel Electrophoresis (BN-PAGE) system. Blue Native PAGE was performed on Cleaver Scientific vs. 10 electrophoresis system, 5–12.5% T gradient gels according to the protocol of Kügler [18]. Tricine-SDS PAGE in reducing conditions for the second dimension were performed on 15% T gels according to Kügler [18], based on [19]. All samples were isolated and gels were run in triplicate.

## 3. Results

### 3.1. Prompt Chlorophyll A Fluorescence

The course of Chl a fluorescence transitions during its characteristic phases O–K, O–J, J–I, and I–P depends on the physiological state of the photosynthesizing object. Changes in the shape of induction curves can be visualized by calculation of differences in fluorescence values between investigated (gall cortex, inner or outer) and control (reference normal photosynthetic plant *A. thaliana*) objects—calculation of difference curves (DC) described by [14,20]. The differences in the IC shapes during the characteristic phases are manifested as specific bands in DC, named L band (between O and K (300 μs)), K band (O–J), H band (J–I), and G band (I–P). We carried out a standard OJIP analysis of the prompt fluorescence induction curves and DC calculations in order to evaluate the photosynthetic activity in *Smicronix* galls on *C. campestris*.

The OJIP fluorescence rise reflects Q_A_ reduction, with Q_A_ equilibrium depending on the poise of the intersystem electron carriers, which, in turn, depends also on the redox state of P700 (PSI reaction center).

**Figure 2:**
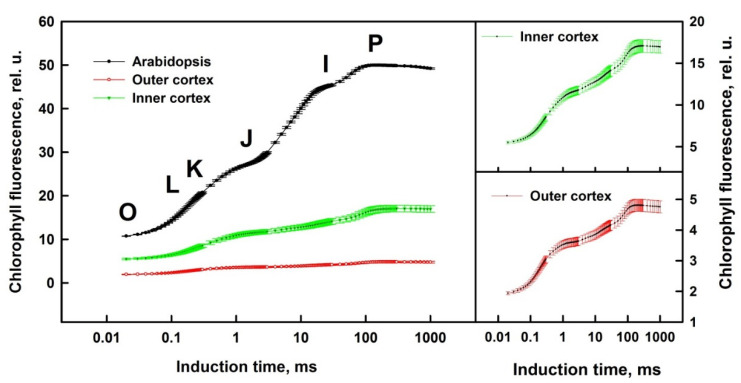
Induction curves recorded in inner and outer cortices of the *Smicronix* galls and in the leaves of reference photosynthetic plant *A. thaliana* (averaged over seven repetitions). The prompt fluorescence of the two cortices of the galls was measured in about 2 mm thick slices. The IC for the inner cortex only is presented above on the right, and for the outer cortex only, below on the right. The ranges of the relative units on the y-axes of the two smaller graphics on the right are different so that the slopes can be visible.

Induction curves showed significantly lower fluorescence of galls, especially of the outer cortex (Figure 2). From the two figures on the right, it can be seen that for the inner cortex F_o_ ≈ 5, F_m_ ≈ 17, i.e., F_v_/F_o_ ≈ 2.5, while for the outer cortex F_o_ ≈ 2, F_m_ ≈ 5, F_v_/F_o_ ≈ 1.5. This means less photosynthetic material in the outer cortex—lower concentration of photosynthesizing structures or a smaller amount of active photosynthesizing structures and photosynthetic membranes.

**Figure 3:**
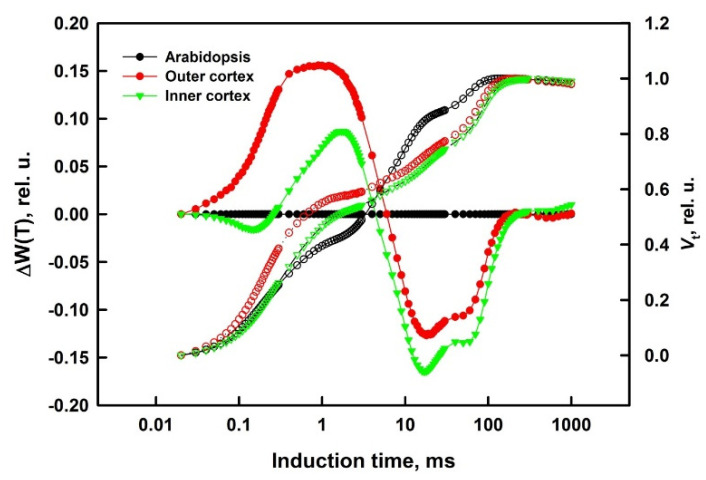
Double normalized curves presenting the relative variable fluorescence V_t_ in O−P transient of chlorophyll fluorescence, recorded in the cortex (inner or outer) of the *Smicronix* galls and in the reference normal photosynthetic plant *A. thaliana* (averaged over seven repetitions). The prompt fluorescence of the two cortices of the galls was measured in about 2 mm thick slices. The values of the relative fluorescence are presented on the right axis (empty symbols).

O, J, I, and P characteristic steps of Chl *a* fluorescence traces were used for calculations of different JIP-test parameters and estimation of structural and functional differences between inner and outer cortex of galls. Comparison of courses of normalized fluorescence traces by calculation of difference curves allows detailed evaluation of structural and functional differences in the investigated objects (Figure 3).

The OJIP fluorescence rise reflects Q_A_ reduction, which depends on the poise of the intersystem electron carriers, which, in turn, is linked with the redox state of PSI reaction centre, P700. The main differences between the IC of gall cortex and the IC of *A. thaliana* leaves were significantly higher levels of the relative variable fluorescence during the initial phases of the transition (between 0 and 10 ms) and a subsequent lag in its increase (10–300 ms) in gall cortices compared to *A. thaliana* (Figure 3). High values for J level indicate lower (or even blocked) electron flow in the acceptor side of PSII, more pronounced in the outer cortex (out).

**Figure 4:**
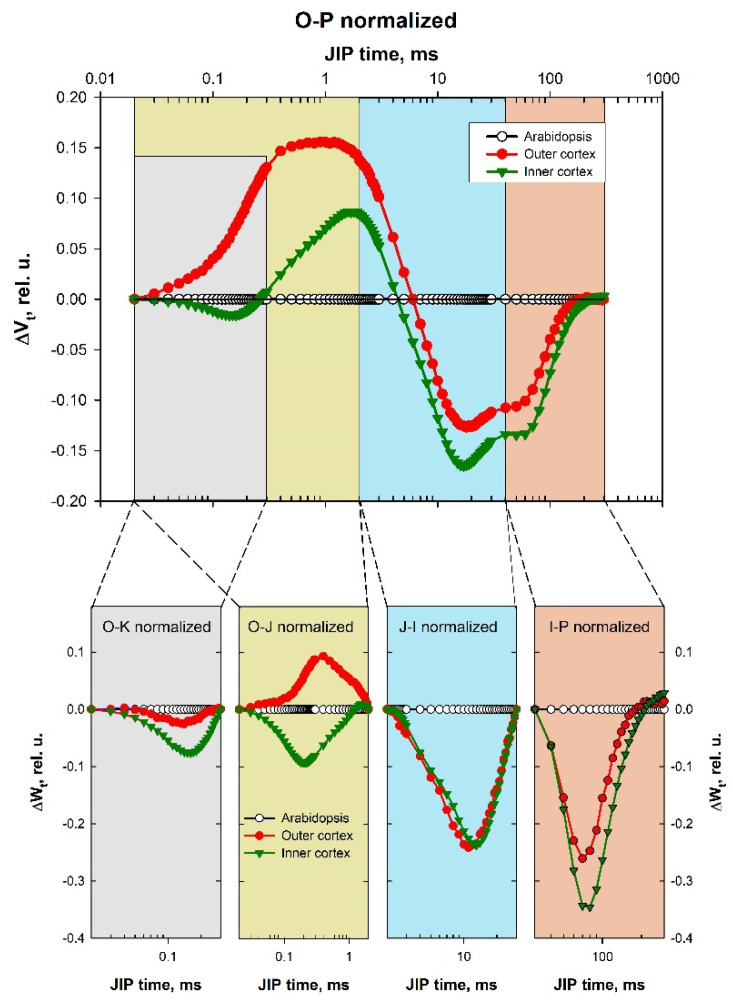
Difference curves showing differences in IC of PF between the gall cortex (inner or outer) and the reference plants *A. thaliana*. Each DC value was calculated as a difference between the values of the relative variable fluorescence [V_t_ = (F_t_ − F_O_)/(F_M_ − F_O_)] in the gall cortex minus the respective values for the leaves of the reference plants *A. thaliana* [ΔV_t_ = V_t(cortex)_ − Vt _(*A. thaliana*)_]. The four characteristic bands are marked with different colors (A). The four lower panels (B–E) represent the difference curves calculated for each band separately (ΔW_t_). The equations for the calculations of the respective ΔW_t_ are presented in Materials and Methods.

Detailed analysis of the structural and functional differences in investigated objects could be done by comparison of the DC, calculated for the characteristic phases of the induction curve. Well-pronounced negative L, H, and G bands were manifested in the difference curves (Figure 4).

***L band (O–K, O–300 μs)***

The functionality of PSII (i.e., antenna size, connectivity between PSII RCs) depends on the pigment-protein complexes packing in the PSA [21]. The L band is associated with the arrangement of PSII units in the thylakoid grana membrane—“grouping”, and with the “connectivity” of LHCII and PSII reaction centers [21,22]. The negative maximums of L bands indicated increasing grouping between neighboring PSII in thylakoid membranes. A negative L peak is formed at a better possibility for energy transfer between adjacent antenna complexes of PSII when the antennae are better packed, or when they are larger and in contact with each other. Positive differences mean ungrouping of the antenna complexes [20]. The negative L-band values are particularly pronounced in the inner cortex (IC).

***K band (O–J, 20 μs–2 ms)***

The K band reflects a photochemical reduction of Q_A_ and partial reoxidation of Q_A_^–^ by PQ (via Q_B_). It gives information about the state of the donor side of PSII—the fraction of operating oxygen-evolving complexes (OEC)—and particularly about Mn-complex in PSII donor side [23,24,25,26]. It is a specific rise with a maximum at about 300 μs on the OJIP induction curve, which is mostly hidden between O and J in normal conditions but is often observed under heat or drought stress [23,27]. The K band characterizes the ratio of the electron transport rates between the donor and acceptor side of PSII and indicates how they operate. The differences in K band could be positive, due to slower electron transport from the donor side toward PSII reaction centers P680^+^ (because of OEC inactivation) and/or faster electron withdrawing from the acceptor side, or negative, because of faster electron transport in the donor side (electron transport acceleration) and/or slower withdrawing of electrons from the acceptor side (electron transport delay).

The only positive peak is the K peak in the outer cortex, which indicates that the electron donation from OEC is slower in the cells of the outer layers as compared to *A. thaliana* plants (Figure 4). The negative values of that band in the inner cortex are a result more of slower electron-withdrawing from the PSII acceptor side than due to the higher rate in the donor side. OEC in the inner cortex works better than the outer one. The negative values for the inner cortex are actually L band, not a K peak. The K peak is not visible. The donor side of the OEC works well, but only the L band of the packaged antenna complexes is revealed. The superposition of the changes in processes leading to L band formation, and not so much the changes in processes connected to K band formation, results in the negative values. Thus, the negative values did not mean that the PSII donor side works properly but revealed some electron transport delay in the PSII acceptor side.

***H band (J–I, 2–40 ms)***

J−I transition reflects the dynamics of the PQ pool reduction between the two PS. H band gives information about the PQ pool volume (number of active PQ molecules functioning between the two PS) and about the electron transfer to Q_A_^–^ and the rate of electron withdrawal from Q_A_^–^ [14,16]. The electron flow to the end acceptors in PSII strongly correlates with the PQ pool size and the degree of its reduction.

When the PQ pool capacity decreases, the rate of reduction is higher, and this results in positive values of the transient H band. Conversely, if the relative size of the PQ pool increases, there will be negative values for that band. The H band represents the dynamics of reduction of PQ pool [16]. Positive differences mean the PQ pool is relatively smaller, and negative means the PQ pool is relatively bigger. Our results showed almost identical negative H bands for both outer and inner cortices of *Smicronix* galls, which means the same increase in PQ quantity in comparison to *A. thaliana* leaves. The relative volume of the pool of PSI acceptors increased.

***G band (I–P, 40–300 ms)***

I–P transition gives information about the PSI functioning and reoxidation of the PQ pool from the PSI carriers. The amplitude of the differences (with a maximum at about 150–200 ms) informs about electron transfer between the pool of reduced PQ and the pool of PSI end acceptors (phylloquinone–FeS cluster–ferredoxin–FNR–NADP^+^) [14] and to the relative PSI content [28]. The efficiency of the electron flow and the rate of the reduction of the PSI end electron acceptors determines the shape of the G band. With a larger end acceptors pool, the rising to the maximum will be slower, and this will result in negative values of the transient band. If the pool of PSI end electron acceptors decreases, the transient will be faster, and a positive peak in the DCs will appear. We observed negative G band values, better pronounced for the inner cortex, which means PSI is more active and the relative volume of the end PSI acceptors pool in both gall cortices is bigger than in *A. thaliana* leaves (Figure 4).

### 3.2. JIP-Test Parameters

**Figure 5:**
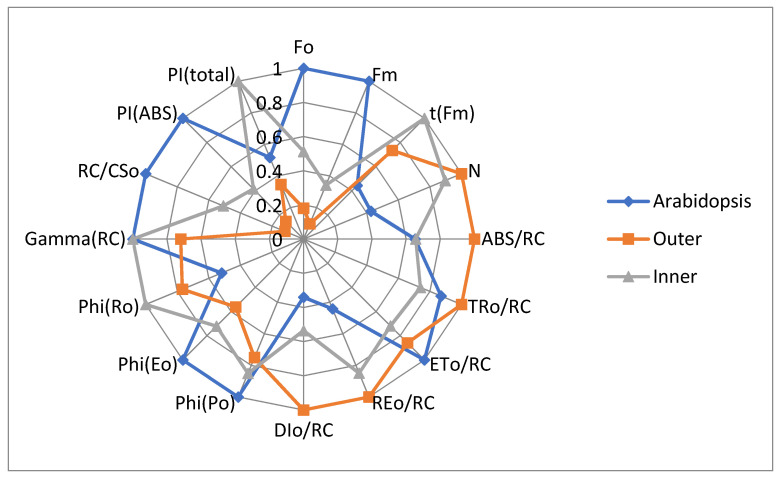
Radar chart of sixteen JIP-parameters calculated from the induction curves of the prompt Chl a fluorescence in outer and inner cortices of *Smicronix* galls and in leaves of the reference normal photosynthetic plant *A. thaliana*. The values of each parameter were normalized to the respective maximum.

JIP-test parameters provide better visualization of the differences between *Smicronix* galls cortices and the reference plant *A. thaliana* (Figure 5).

Lower values of minimal and maximal fluorescence (parameters F_o_ and F_m_) in both inner and outer cortices in comparison to the *A. thaliana* leaves are a result of a low density of photosynthetic structures (RC/CSo). The number of RC is especially low in the outer cortex. N, the number of Q_A_ redox turnovers until FM is reached, characterizes the relative size of PQ pool per reaction center. In both cortices, N is bigger than in *A. thaliana* leaves and a bit smaller in the inner cortex than in the outer; there are PQ molecules, but reaction centers are few.

ABS/RC characterizes the size of the PSII antenna complexes. It is bigger in the outer cortex than in the inner one or in *A. thaliana* leaves. Having bigger antenna complexes in the outer cortex, we can expect more effective energy trapping, but the values of the parameter TR_0_/RC for outer cortex are close to that for *A. thaliana*. Obviously, the energy transfer in the *A. thaliana* PSII antennae is much more effective than in the outer cortex, and especially more effective than in the inner cortex. With a smaller antenna in *A. thaliana*, the efficiency of energy transfer is better than in *Smicronix* gall cortices. Lower values of ETo/RC in gall cortices indicated that the acceptor side in both cortices functions worse than in *A. thaliana* leaves.

Unlike PSII, PSI works more efficiently in gall cortices (especially in the outer cortex) than in *A. thaliana*. *Arabidopsis* values for REo/RC (electron flux reducing end electron acceptors at the PSI acceptor side per PSII reaction center) are much lower than those of *Smicronix* gall cortices. In general, the energy transfer to RC in gall cortices significantly exceeded the energy transfer in *A. thaliana*; i.e., the PSA in the reference normal photosynthetic plant operates optimally but is not optimized in *Smicronix* galls. A lot of excitation energy in gall cortices was dissipated as heat in PSII antennae and reaction centers, especially in the outer cortex (see DI_0_/RC on Figure 5).

The parameter φ_Eo_, the quantum yield for electron transport, shows that the PSII RC worked effectively in the three investigated samples, with maximal efficiency in Arabidopsis leaves, and lower in gall cortices. The differences in φ_Po_, the maximum quantum yield of primary photochemical reactions in PSII RC, are similar to that for φ_Eo_ but more pronounced for φ_Eo_. The three samples differ in the efficiency of the PSII acceptor side. The differences in the quantum yield of the electron transport from Q_A_^–^ to the end electron acceptors of PSI, φ_Ro_, also indicated that PSI operates more effectively in cortices than in *A. thaliana*, and with better effectivity in the inner than in the outer cortex. The energy transformation effectiveness in PSI is almost the same in the two cortices (a bit bigger in the inner cortex) and about 2.5 times lower in *A. thaliana* (manifested by parameter δ_Ro_, efficiency for an electron to move from reduced carriers between the two photosystems to the end acceptors of PSI, δ_Ro_ = RE_0_/ET_0_, data not shown). This high efficiency compensates the energy losses in PSII, and the φ_Ro_ values are the biggest in the inner cortex and lowest in *A. thaliana*.

The density of active PSII reaction centers per cross section (RC/CSo) is very low in both cortices, especially in the outer one. In comparison to *A. thaliana* leaves, it is two times lower in the inner cortex, and about ten times lower in the outer cortex. This affects the PSII efficiency in gall cortices, and their PI_ABS_ is very low. PI_total_ value in the inner cortex is maximal due to significant compensation from the high efficiency of PSI. In general, the PSA in *Smicronix* gall cortices is poorly developed quantitatively and qualitatively; not only is the number of structures smaller compared to normal photosynthetic leaves, but also the electron transport chain in galls does not work effectively with regard to PSII. PSI is well developed and works with quite high efficiency. The inner cortex has a higher chlorophyll concentration, and it is photosynthetically more active.

### 3.3. Thylakoid Complexes

**Figure 6:**
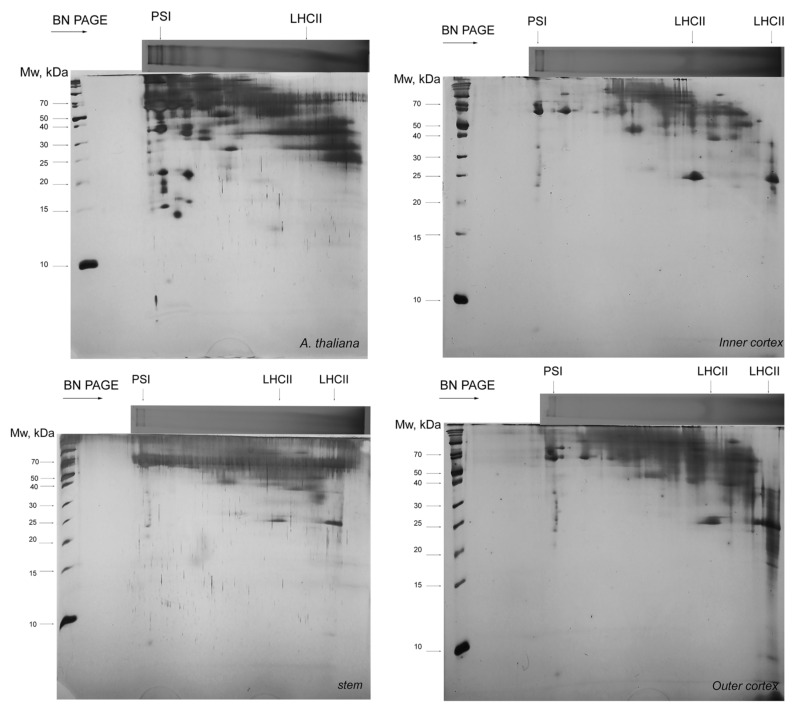
2D BN/SDS PAGE separation of thylakoid complexes in *Arabidopsis thaliana*, *Cuscuta campestris* stem, and *Smicronyx* galls (inner and outer cortex, respectively). Solubilization with 1% digitonin. Only the low-molecular-weight standards are indicated.

To further elucidate the photosynthetic machinery of the *Smicronyx* galls, we performed solubilization with digitonin and two-dimensional electrophoretic separation (BN PAGE as first dimension, and 15% T Tricine SDS PAGE as the second dimension). The obtained results are shown in Figure 6. Identification of complexes is based on the comparison to already-published molecular weight of the proteins within the subcomplexes [9] and 2D profiles [17]. Obviously, the number of complexes is fewer in *C. campestris* than in *A. thaliana* (Figure 6), but on the other hand, the differences between the inner and outer cortices and the non-photosynthetic stem are not substantial and may differ mainly in the abundance of particular complexes. At least PSI and LHCII were found in *C. campestris*, but most of the other complexes, identified in *A. thaliana*, are absent.

## 4. Discussion

There are a few investigations in the available literature [11] showing that *Smicronix* galls have higher Chl concentration and higher photosynthetic activity in comparison with the *Cuscuta* stem, and the highest Chl content and the most highly developed chloroplasts were observed in the tissue around the larval chamber. This is probably related to the increased need for nutrients for the developed larvae. The fact the galls have a green color and photosynthetic activity until the larvae are alive supports this hypothesis [11]. When the larva developed and mature weevils left the gall or when larva died (e.g., with *Bracon murgabensi* infection), the photosynthesis in galls ends. Anikin et al. [11] investigated some parameters of PSII fluorescence in *Smicronyx smreczynskii* galls on *C. campestris* and found an increased quantity of active PSII RCs and increased effectiveness of photosynthesis in the galls compared to *C. campestris* stems. We further expand this knowledge by studying in detail the parameters of chlorophyll fluorescence by employing the JIP-test (Figure 2, Figure 3 and Figure 4).

The normal Chl a/b ratio in green plants is about 3. The photosystem II contains three types of light-harvesting complexes: the core antennae containing Chl a only and most closely associated with the reaction center; the minor Chl a/b binding proteins, which occupy an intermediate position between the core antennae and the peripheral antenna; and the outermost Chl a/b binding proteins, LHCII [29] and references therein. These three antenna groups account for 14, 15, and 67%, respectively, of the total chlorophyll in PSII; see [30] and references therein. LHCII is the major component of the thylakoid membrane and the major light-harvesting antenna of PSII, with Chl a/b ratio close to 1 (8:7 molecules). In addition to harvesting light, LHCII plays a role in membrane stacking, which has consequences in the regulation of the distribution of energy between the two photosystems. The normal Chl a/b ratio in PSI is 5–6. In a previous experiment, we have observed a significant increase in both chlorophyll a and chlorophyll b concentrations in the *Smicronix* gall cortices in comparison to the *C. campestris* stem [12]. Chl a content increased almost twofold in the outer cortex and over threefold in the inner cortex of the gall. Chlorophyll b was not detected in the non-infected stems, and it was in the highest concentration in the inner cortex. Chl a/b ratio was about 5–6 in the cortices. These results provide indirect evidence that PSI is better organized in galls. Our results from the electrophoretic separation of thylakoid complexes showed much less PSI and LHCII in galls compared to *A. thaliana* (Figure 6). Unfortunately, with that method, we cannot identify other complexes. The LHCII shortage could result in lesser energy flow toward PSII RC in galls than in *A. thaliana*. Other authors were also observed low PSII efficiency in *C. campestris* [8,31]. Sherman et al. [32] found that the *C. pentagona* chloroplast contains a number of the proteins required for a successful fixation of CO_2_ and the proteins in the thylakoids are organized much like other higher plants, with the exception of the large percentage of the thylakoids organized into grana.

As we demonstrated, *C. campestris*, under certain circumstances (i.e., in the *Smicronyx* galls) is able to properly arrange its photosynthetic machinery and to perform sufficient photosynthesis (Figure 2). This is not an exception in nature. Many plants are able to turn heterotrophic organs (roots) green. It is considered to contribute to the carbon economy of the whole plant ([33] and references therein). *A. thaliana* roots also have the potential to synthetize chlorophyll and to develop chloroplasts under light, with phytohormone signaling involved. The process is more effective when the shoots have been removed. The O-J-I-P transients were observed in root samples, indicating the presence of functional electron transport in PSII. The PSII efficiency was lower than in the shoot chloroplasts, and the electron transport from Q_A_^–^ to Q_B_ was slowed down in the root plastids. The roots demonstrate accelerated chlorophyll accumulation and chloroplast development when they are detached from shoots. The authors concluded that the increased photosynthesis by “green roots” could contribute to the carbon economy of the whole plant [33]. The induction of chlorophyll synthesis and chloroplasts development in infected *C. campestris* plants might be related to the increased need for carbohydrates for the *Smicronyx* larvae nutrition [12]. The major differences, predetermining the increased and more efficient photosynthesis in the galls as compared to the stem are not in the thylakoid complexes (Figure 6) but probably in the chlorophyll concentration [12]. It was reported that most of the photosynthetic proteins are present in *C. campestris*, at least at a gene level [6], but still, they are underrepresented even in the inner gall cortex as compared to *Arabidopsis thaliana*.

Photosynthesis may vary significantly between *Cuscuta* species [5,34,35], but *C. campestris* is among the better photosynthetizers in the genus [8]. This capacity may further vary depending on environmental factors, including the presence of *Smicronyx* galls. Its inability to function as an independent/self-reliant plant is a result of the non-functional roots [6,32,36], but also of the lack of developed leaves, which limits the surface available for light absorption. Most importantly, the stem of *Cuscuta* spp., and *C. campestris* stem in particular, has low chlorophyll concentration [12,34] but is very rich in carotenoids, especially lutein-5, 6-epoxide, and 9-cis-violaxanthin [37,38], which is important in photoprotection. This might be also beneficial for the *Smicronyx* larvae, ensuring its protection from high light intensity by the outer cortex of the gall.

However, is the formation of the gall detrimental or beneficial to the parasitic plant? Some authors suggest a new view of the relationship between *Smicronyx* and *Cuscuta*—not epiparasitism, but a mutually beneficial interaction between the species [11]. Although the impact of the insect larvae did not seem to be negative in the gall itself—photosynthesis is enhanced and no significant oxidative stress was found [12], it may be negative at the individual plant level, as other reports showed the galls could restrict the flow of nutrients to the flowers of *Cuscuta*, thus limiting the number of viable seeds [39].

## 5. Conclusions

The infection of *Smicronyx* on *C. campestris* leads to the development of galls with morphologically and functionally distinct inner and outer cortices. Our results showed some photosynthetic activity in the gall cortices, which were more effective in the inner one. It is a result mainly of increased chlorophyll *a* concentration rather than qualitative changes in the thylakoid complexes. No apparent differences were found in the number of identified thylakoid complexes between the gall cortices and *C. campestris* stem, although the abundance in the stem seemed to be lower. The higher chlorophyll concentration in the inner cortex and the higher photosynthetic activity are related to its main function as a direct nutritional source for the larvae. The outer cortex probably has a more protective function. We consider the insect-triggered transition from non-photosynthetic to photosynthetic tissue in the gall to be driven by increased nutrient requirements associated with larval feeding. This induced photosynthesis did not reach the levels of the photosynthetic activity in *Arabidopsis thaliana* plants.

## Data Availability

Not applicable.

## Abbreviations

Chl: chlorophyll; DC: different curves; IC: induction curves; OEC: oxygen-evolving complex; PF—prompt chlorophyll *a* fluorescence; PSA: photosynthetic apparatus; PS (I, II): photosystem (I, II), PQ: plastoquinone, RC: reaction center.

## Appendix A

**Table A1:** Definition of selected OJIP parameters according to Strasser et al. [14] and Stirbet and Govindjee [15].

Parameter	Definition
F_O_	Minimal fluorescence, when all PSII reaction centers (RCs) are open, fluorescence intensity at 20th µs
F_M_ = F_P_	Maximal fluorescence recorded under saturating illumination at the peak P of OJIP, when all PSII RCs are closed
F_J_ (2 ms)	Fluorescence level at 2nd ms, the J-step of OJIP
F_I_ (40 ms)	Fluorescence level at 40th ms, the I-step of OJIP нa гpaφикaтa e 40-тa ms
F_V_	Maximal variable fluorescence, F_V_ = F_M_ − F_O_
V_t_	Relative variable fluorescence at time *t*: V_t_ = (F_t_ − F_O_)/(F_M_ − F_O_)
V_L_	Relative fluorescence value at L-step [0.15 ms]
V_K_	Relative fluorescence value at K-step [0.3 ms]
N	Number indicating how many times Q_A_ is reduced until fluorescence reaches its maximal value F_M_ (number of Q_A_ redox turnovers until F_M_ is reached)
ABS	Absorbed energy flux (exited PSII antennae Chl *a* molecules)
TR_0_	Trapped energy, i.e., energy, utilized for reduction of pheophytin and primary quinone acceptor Q_A_
ET_0_	Electron transport from Q_A_^–^ to the next electron acceptors between the two photosystems.
RE_0_	Energy/electron flux for reduction of end acceptors in acceptor side of PSI—NADP and ferredoxin.
φ_Eo_ (ET_0_/ABS)	Efficiency/probability for the electron in PSII to move further than Q_A_^–^—quantum yield for electron transport. φ_Eo_ = ET_0_/ABS = φ_Po_ × ψ_0_ = (1 − F_0_/F_M_) × ψ_0_
ABS/RC	Absorbed energy flux in antenna chlorophylls per PSII reaction center (a measure of PSII apparent antenna size)
TR_0_/RC	Trapped energy flux per RC; TR_0_/RC = M_0_ (1/V_J_)
ET_0_/RC	Electron transport flux further than Q_A_^–^ per PSII reaction center
RE_0_/RC	Electron flux reducing end electron acceptors at the PSI acceptor side, per PSII reaction center
DI_0_/RC	Heat dissipation of excitation energy by PSII reaction center
ET_0_/RC	Electron transport flux further than Q_A_^–^ per PSII reaction center
φ_Po_ (TR_0_/ABS)	Maximum quantum yield of primary photochemical reactions in PSII RC. φ_Po_ = TR_0_/ABS = 1 − F_O_/F_M =_ F_V_/F_M_
φ_Ro_ (RE_0_/ABS)	Quantum yield of the electron transport from Q_A_^–^ to the end electron acceptors of PSI RE_0_/ABS = φ_Ro_ = φ_Po_ × ψ_0_ × δ_0_
RC/CS_0_	Density of active PSII reaction centers (RC) per cross section RC/CS_0_ = φ_Po_ × (V_J_/M_0_) × (ABS/CS_0_)
PI_ABS_	Performance index (potential) for conservation of the energy absorbed by PSII RC until the reduction of intersystem electron acceptors
PI_total_	Performance index (potential) for conservation of the energy absorbed by PSII until the reduction of PSI end electron acceptors
